# Atlantoaxial Subluxation Associated With Chronic Motor Tics

**DOI:** 10.7759/cureus.37543

**Published:** 2023-04-13

**Authors:** Miho Ando, Toru Funayama, Kotaro Sakashita, Tomoyuki Asada, Masashi Yamazaki

**Affiliations:** 1 Department of Orthopaedic Surgery, Institute of Medicine, University of Tsukuba, Tsukuba, JPN

**Keywords:** atlantoaxial subluxation, chronic motor tics, high cervical myelopathy, ponticulus postics, c1 lateral mass screw

## Abstract

Head jerking is one of the most common symptoms of motor tics, and because of this, patients are at an increased risk of cervical spine disorders. However, there have been no reports of atlantoaxial subluxation in the English literature. To the best of our knowledge, this is the first case of atlantoaxial subluxation associated with chronic motor tics. A 41-year-old man with a history of chronic motor tics since childhood was diagnosed with high cervical myelopathy due to atlantoaxial subluxation. The patient underwent posterior fusion surgery using atlantoaxial instrumentation and an autologous bone graft. Although screw breakage occurred as an early postoperative instrumentation failure, the clinical outcome was excellent after surgery without recurrence of subluxation. Other techniques such as atlantoaxial transarticular fixation and occipitocervical fusion followed by long-term external immobilization might be treatment options at the initial surgery, or in case of postoperative recurrent atlantoaxial subluxation.

## Introduction

The Diagnostic and Statistical Manual of Mental Disorders, Fifth Edition, defines tics as “sudden, rapid, recurrent, nonrhythmic motor movements or vocalizations, generally preceded by urge”, whereas the diagnosis of Tourette syndrome requires the presence of both chronic motor tics with chronic phonic tics [1]. The estimated prevalence of Tourette syndrome is 0.05% in adults [2]. Because head jerking is one of the most common symptoms of motor tics [1], patients are at an increased risk of cervical spine disorders [3]. To date, 16 cases of spondylotic myelopathy at the subaxial cervical levels have been reported [4]. However, there have been no reports of atlantoaxial subluxation (AAS) in the English literature. Here, we report the first case of high cervical myelopathy due to AAS associated with chronic motor tics.

## Case presentation

A 41-year-old man with a history of chronic motor tics since childhood presented to our hospital complaining of posterior neck pain and disturbance of bilateral hand coordination with numbness lasting three months. He was engaged in electrical work, which often required the adoption of an unnatural neck position in a narrow space. At the initial visit, head jerking was observed every 10-15 seconds. He had no phonic tics. Scapulohumeral reflex (Shimizu) was bilaterally positive. Reflexes of the left triceps tendon, bilateral patellar tendon, and bilateral Achilles tendon were increased. His grip power was 33 kg in the right and 24 kg in the left. The Japanese Orthopaedic Association (JOA) score of cervical myelopathy was 14.5/17 points.

**Figure 1:**
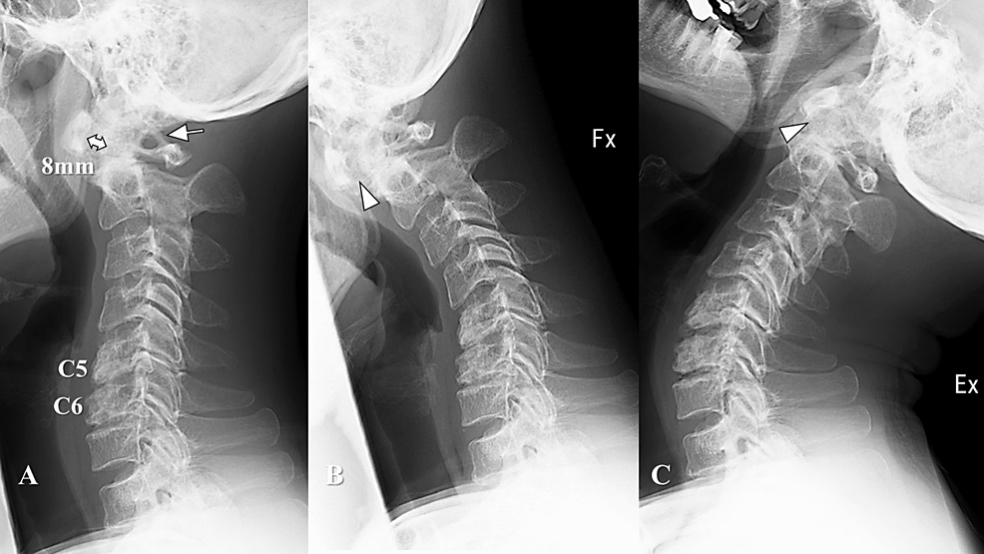
Radiography images at the initial visit. A. Lateral plain radiography in standing position revealed atlantoaxial subluxation with an 8-mm atlantodental distance (double arrow) and ponticulus postics (arrow). Slight spondylotic changes were further observed at the C5/6 and C6/7 spinal levels. B and C. Horizontal atlantoaxial instability during neck flexion (B) and extension (C) was observed (arrowheads).

**Figure 2:**
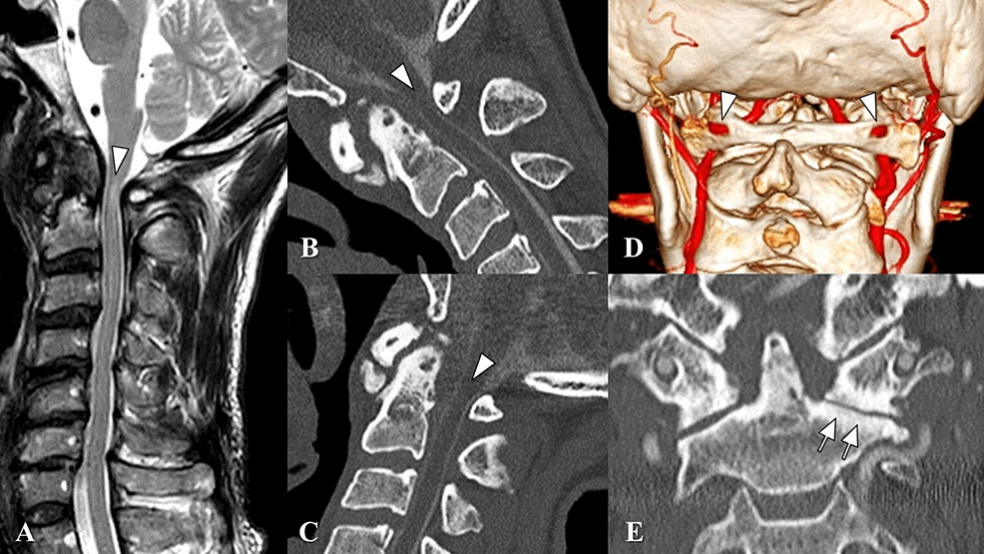
Preoperative images. A. T2-weighted magnetic resonance imaging (MRI) revealed severe spinal cord compression with a high signal change at the C1 spinal level (arrowhead). B and C. CT myelography showed that spinal cord compression was exacerbated during flexion (B; arrowhead) and released during extension (C; arrowhead). D. Three-dimensional CT angiography revealed that bilateral vertebral arteries (VA) ran through the ponticulus postics (arrowheads). E. Osteoarthritis at the left C1/2 facet joint was further observed (arrows) in the coronal CT scan.

Lateral plain radiography revealed AAS with ponticulus postics (PP) and slight spondylotic changes at the subaxial cervical levels (Figure 1A). Horizontal instability of AAS was observed (Figure 1B-1C). T2-weighted magnetic resonance imaging (MRI) revealed spinal cord compression (Figure 2A). Computed tomography (CT) myelography showed that spinal cord compression was exacerbated during flexion (Figure 2B) and released during extension (Figure 2C). CT angiography revealed that the bilateral vertebral arteries (VA) ran through the PP (Figure 2D). Osteoarthritis of the left C1/2 facet joint was further observed (Figure 2E).

**Figure 3:**
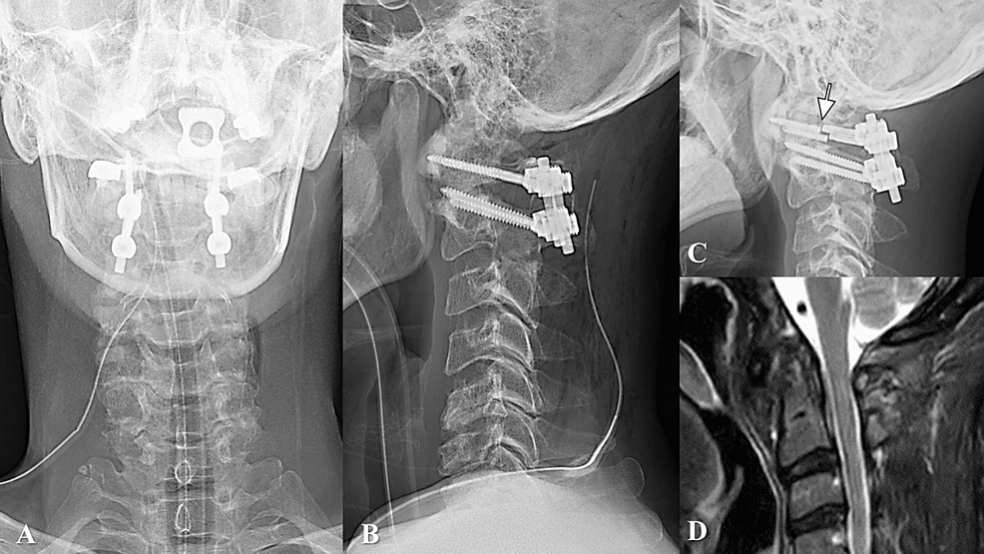
Postoperative images. A and B (plain radiography immediately after surgery). The patient underwent posterior atlantoaxial fusion surgery using C1 lateral mass screws (LMS) and C2 pedicle screws. Due to the bilateral ponticulus postics, C1 LMS were inserted inferior to the posterior arch to avoid vertebral arteries (VA) injury. All screws were inserted bicortically for stronger fixation. After instrumentation, an autologous iliac bone block was grafted between the C1 posterior arch and the C2 spinous process. C (three months post surgery). Although lateral plain radiography showed bilateral C1 LMS breakage (arrow), atlantoaxial subluxation did not recur. D (four months post surgery). Recurrent spinal cord compression was not detected in the T2-weighted MRI.

The patient underwent posterior fusion surgery with a previously reported method [5] (Figure 3A-3B). Due to the bilateral PP, C1 lateral mass screws (LMS), 3.5 mm in diameter and 32mm in length, were inserted inferior to the posterior arch [5] to avoid VA injury [6]. All screws were inserted bicortically for stronger fixation [5]. All implants, including rods, were made of titanium alloy. After instrumentation, an autologous iliac bone block was grafted between the posterior arch of C1 and the spinous process of C2.

**Figure 4:**
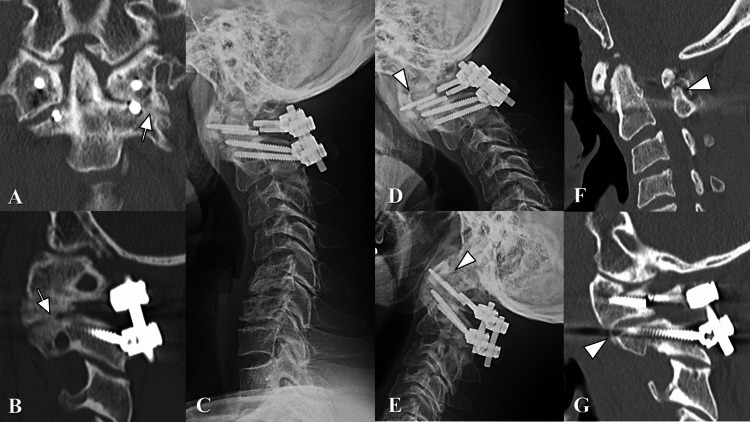
Postoperative images (continued). A and B (six months after surgery). Spontaneous fusion was detected at the left C1/2 facet joint (arrows) that originally had osteoarthritis in the coronal (A) and the sagittal (B) CT scan. C (12 months after surgery). Lateral plain radiography in standing position revealed kyphotic change at the subaxial cervical spine, which was already seen with spondylotic changes preoperatively, progressed slightly. D and E (12 months after surgery). Atlantoaxial interval did not change during neck flexion (D) and extension (E) (arrowheads). F and G (12 months after surgery). Although bony union was not observed between the posterior arch of C1 and the spinous process of C2 (F), further spontaneous fusion was detected at the right C1/2 facet joint by bridging of the anterior bony spur (G) in the sagittal CT scan.

After surgery, he wore a neck soft collar for six months. His hand coordination improved with occupational therapy as rehabilitation, and he returned to work four months after surgery. At six months postoperatively, the grip power improved to 44 kg in the right and 35 kg in the left. The JOA score had improved to 16. Although bilateral C1 LMS broke three months after surgery, AAS did not recur (Figure 3C), and recurrent spinal cord compression was not detected (Figure 3D). Spontaneous fusion was detected at the left C1/2 facet joint that originally had osteoarthritis six months postoperatively (Figure 4A-4B). Kyphotic change at the subaxial level progressed slightly 12 months postoperatively (Figure 4C), whereas atlantoaxial instability did not recur (Figure 4D-4E). Although bony union was not observed between the posterior arch of C1 and the spinous process of C2 (Figure 4F), further spontaneous fusion was detected at the right C1/2 facet joint by bridging of the anterior bony spur (Figure 4G). As of 16 months after surgery, the patient had no complaints of posterior neck pain and no recurrence of myelopathy.

## Discussion

The stability of the atlantoaxial joint is maintained by not only the transverse ligament but also the fibrous joint capsules, which work as a whole [7]. In our case, unilateral C1/2 facet joint osteoarthritis was already observed preoperatively. The pathogenesis was considered as follows: chronic motor tics had exerted long-term minor mechanical stress on the soft tissues of the atlantoaxial structure causing AAS.

In our case, the clinical outcome was excellent without recurrence of atlantoaxial subluxation, whereas bilateral C1 LMS breakage occurred as an early postoperative instrumentation failure. It was considered that continuous mechanical stress due to chronic motor tics on the fixed atlantoaxial joint caused screw breakage. Although there was a potential risk of VA damage by the broken screws, no vascular events happened. Fortunately, the unilateral C1/2 facet joint had osteoarthritis preoperatively, so it is considered that spontaneous fusion of the facet joint was achieved by sufficient fixation of the atlantoaxial joint until the screw breakage. As a result, a recurrence of atlantoaxial subluxation did not occur.

Other techniques such as atlantoaxial transarticular fixation or reinforcement options including hooks and sublaminar tape wiring are all potential options [8]. Further, an occipitocervical fusion followed by long-term external immobilization such as a halo vest might be a treatment option at the initial surgery of the present case, or in the case of postoperative recurrent atlantoaxial subluxation. However, we thought that there was no need to add these potential options or choose an occipitocervical fusion. Due to the first case, there was no credible evidence for adequate fixation strength for the present case. Botulinum toxin was not used in our case because a global consensus for chronic tics has not yet been reached [9]. However, it has been reported to be effective in treating cervical myelopathy associated with motor tics [10]. It may be an option to prevent implant failure. Finally, since subaxial cervical spondylosis progressed slowly, careful long-term follow-up is necessary.

## Conclusions

This is the first case of atlantoaxial subluxation associated with chronic motor tics. Although screw breakage occurred as an early postoperative instrumentation failure, the clinical outcome was excellent after atlantoaxial posterior fusion surgery without recurrence of subluxation.

## References

[1] McGuire JF, Nyirabahizi E, Kircanski K (2013). A cluster analysis of tic symptoms in children and adults with Tourette syndrome: clinical correlates and treatment outcome. Psychiatry Res.

[2] Knight T, Steeves T, Day L, Lowerison M, Jette N, Pringsheim T (2012). Prevalence of tic disorders: a systematic review and meta-analysis. Pediatr Neurol.

[3] Isung J, Isomura K, Larsson H, Sidorchuk A, Fernández de la Cruz L, Mataix-Cols D (2021). Association of Tourette syndrome and chronic tic disorder with cervical spine disorders and related neurological complications. JAMA Neurol.

[4] Ko DY, Kim SK, Chae JH, Wang KC, Phi JH (2013). Cervical spondylotic myelopathy caused by violent motor tics in a child with Tourette syndrome. Childs Nerv Syst.

[5] Harms J, Melcher RP (2001). Posterior C1-C2 fusion with polyaxial screw and rod fixation. Spine (Phila Pa 1976).

[6] Arslan D, Ozer MA, Govsa F, Kıtıs O (2018). The ponticulus posticus as risk factor for screw insertion into the first cervical lateral mass. World Neurosurg.

[7] Iizuka H, Iizuka Y, Kobayashi R (2013). Characteristics of idiopathic atlanto-axial subluxation: a comparative radiographic study in patients with an idiopathic etiology and those with rheumatoid arthritis. Eur Spine J.

[8] Huang DG, Hao DJ, He BR (2015). Posterior atlantoaxial fixation: a review of all techniques. Spine J.

[9] Pandey S, Srivanitchapoom P, Kirubakaran R, Berman BD (2018). Botulinum toxin for motor and phonic tics in Tourette's syndrome. Cochrane Database Syst Rev.

[10] Aguirregomozcorta M, Pagonabarraga J, Diaz-Manera J, Pascual-Sedano B, Gironell A, Kulisevsky J (2008). Efficacy of botulinum toxin in severe Tourette syndrome with dystonic tics involving the neck. Parkinsonism Relat Disord.

